# Toxic Epidermal Necrolysis in a Critically Ill African American Woman: A Case Report Written With ChatGPT Assistance

**DOI:** 10.7759/cureus.35742

**Published:** 2023-03-03

**Authors:** Rebekah Lantz

**Affiliations:** 1 Medicine, Miami Valley Hospital, Dayton, USA

**Keywords:** chatgpt, dermatologic drug reaction, drug rash, skin of color, toxic epidermal necrolysis (ten), stevens-johnson syndrome (sjs)

## Abstract

Stevens-Johnson syndrome (SJS) and toxic epidermal necrolysis (TEN) are life-threatening spectrum diseases in which a medication triggers a mucocutaneous reaction associated with severe necrosis and loss of epidermal integrity. The disease has a high mortality rate that can be assessed by dermatology scoring scales based on an affected total body surface area (TBSA). Sloughing of <10% TBSA is considered SJS, with a mortality of 10%. Sloughing of >30% TBSA is termed TEN, with an increased mortality rate of 25% to 35%. We present a case and management of TEN that involved >30% TBSA in a critically ill African American woman. Identification of the offending agent was difficult due to complicated medication exposure throughout her multi-facility care management. This case conveys the importance of close monitoring of a critically ill patient during a clinical course involving SJS-/TEN-inducing drugs. We also discuss the potential increased risks for SJS/TEN in the African American population due to genetic or epigenetic predispositions to skin conditions. This case report also contributes to increasing skin of color representation in the current literature. Additionally, we discuss the use of Chat Generative Pre-trained Transformer (ChatGPT, OpenAI LP, OpenAI Inc., San Francisco, CA, USA) and list its benefits and errors.

## Introduction

Stevens-Johnson syndrome (SJS) and toxic epidermal necrolysis (TEN) are life-threatening spectrum diseases where infection, malignancy, or, most commonly, medications trigger a mucocutaneous reaction associated with severe necrosis and loss of epidermal integrity. The disease has a high mortality rate that can be assessed by dermatology scoring based on an affected total body surface area (TBSA). Epidermal detachment of <10% is defined as SJS, 10% to 30% is defined as SJS-TEN overlap, and in the case of our patient, ≥30% is defined as TEN [1]. TEN initially presents cutaneously as a confluent erythematous macular rash with dusky centers along the trunk and extremities. As the disease progresses rapidly, it is accompanied by flaccid bullae on the skin and hemorrhagic erosions on involved mucosal surfaces [1]. Dermatologic signs, including the Nikolsky sign and Asboe-Hansen sign, are highly suggestive of TEN/SJS in the setting of dusky erythematous macules and mucosal erosions [2]. TEN can occur in all genders, ages, races, and ethnicity; however, it is more common in women, patients aged 40 years or older and immunocompromised individuals [3]. Certain human leukocyte antigen (HLA) genotypes increase the risk of developing SJS/TEN in various ethnicities, including African Americans [4]. In addition to a possible related genetic component, recent studies have indicated that an increasing number of chronic conditions, infections, hematologic malignancy, and renal failure are associated with significantly higher mortality rates, with the strongest predictor for the length of hospital stay being many chronic conditions (*P* < 0.05) [4].

Our case presents a critically ill African American woman with a complex profile of medical conditions and medication history that ultimately led to a diagnosis of TEN. We emphasize the established racial disparities and risk factors implicated in this case, including possible genetic or epigenetic predispositions to skin conditions in the African American population. We also highlight the challenge of recognizing life-threatening dermatologic pathology in a critically ill patient receiving polypharmacy.

## Case presentation

A 70-year-old African American woman with a history of atopic dermatitis managed with phototherapy, dupilumab, and other immunosuppressants presented with a long and complicated clinical course that resulted in the development of TEN. During her first admission for a right breast hematoma at Hospital-1, her hematoma was evacuated and she received empiric piperacillin/tazobactam for infection, furosemide for edema, and methylprednisolone for inflammation. She was discharged with doxycycline, cephalexin, and prednisone. The patient was transferred to Hospital-2 due to hematoma expansion and was subsequently evacuated again. At this time, her doxycycline and cephalexin were continued and triamcinolone/hydrocortisone was added. She then developed a deep vein thrombosis in her left lower extremity, which was managed with an inferior vena cava (IVC) filter due to recent surgery and relative contraindication to anticoagulation. In addition, ulcerations were noted in her groin, and she was positive for herpes simplex virus 2 (HSV 2). She was treated with valacyclovir and discharged.

In the following weeks, the patient presented to Hospital-3 with sepsis with methicillin-sensitive Staphylococcus aureus (MSSA) bacteremia. Her breast hematoma was evacuated a third time, which grew Pseudomonas in drainage culture. Empiric linezolid was de-escalated to cefepime by the recommendation of an Infectious Disease specialist. She was discharged with intravenous cefazolin via a peripherally inserted central catheter (PICC) for six weeks to a skilled nursing facility (SNF). The Pseudomonas had been considered treated with abscess evacuation by the Infectious Disease specialist. Upon arrival at the SNF, the PICC line was noted to be bleeding, and she was readmitted to Hospital-3 with a supratherapeutic international normalized ratio (INR) of 11.1, notable encephalopathy, and hypokalemia. The patient's family took her home after five days of hospitalization before the finalization of treatment without perceived improvement.

**Figure 1:**
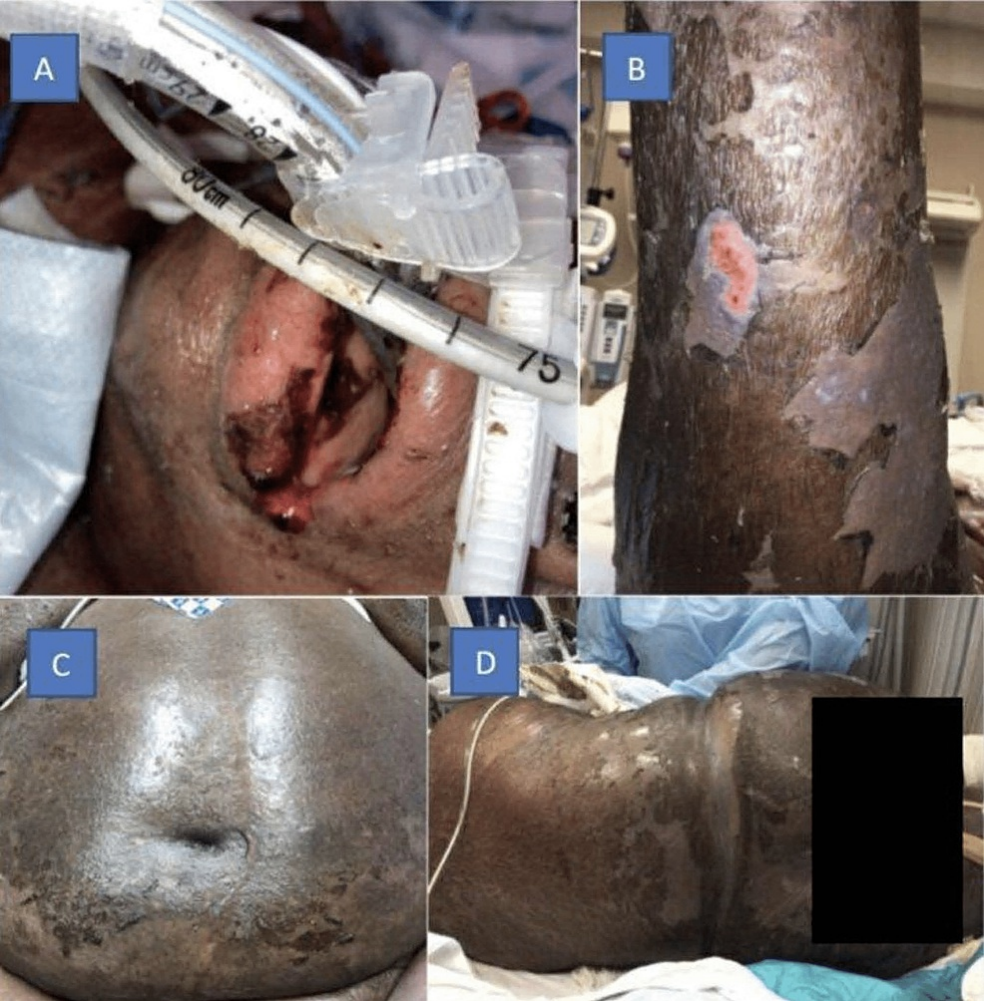
(A) Oral mucosa sloughing; (B) sloughing of right posterior leg and violaceous erythema; (C) abdominal skin sloughing; and (D) area of back involvement.

**Figure 2:**
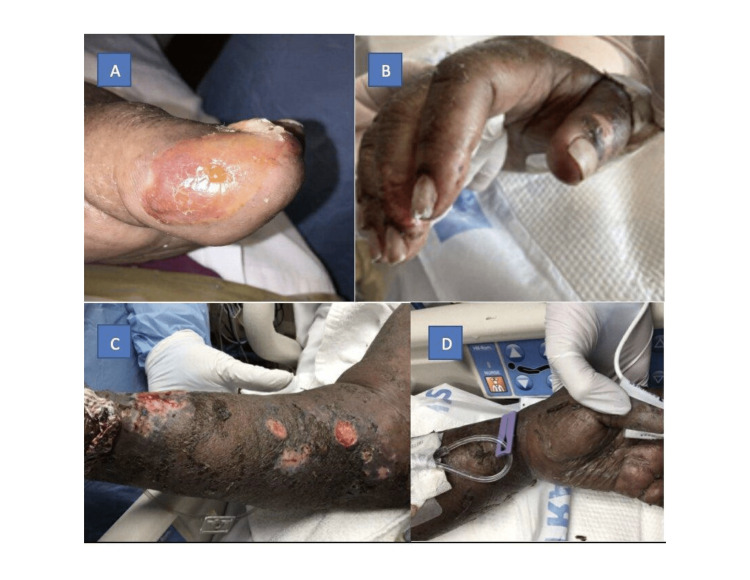
(A) Left hallux bullae; (B) right phalanges sloughing; (C) right forearm ruptured bullae; and (D) left-hand sloughing.

The patient presented back to the emergency department shortly thereafter with septic shock with associated bacteremia and fungemia, respiratory failure, and worsening leukocytosis for which she received vasopressors, shock steroids, and mechanical ventilation with an eventual tracheostomy. She experienced renal failure and required hemodialysis. When blood cultures grew Candida, antimicrobial coverage was broadened to meropenem, daptomycin, and fluconazole. Given new onset seizure-like activity, lacosamide and phenobarbital were added. At this time, nursing noted mucosal and tip-of-digit denudation in the patient. A full skin exam ensued, and lesions are shown in Figures 1-2. Dermatology was consulted due to concern for a severe cutaneous adverse drug reaction.

**Figure 3:**
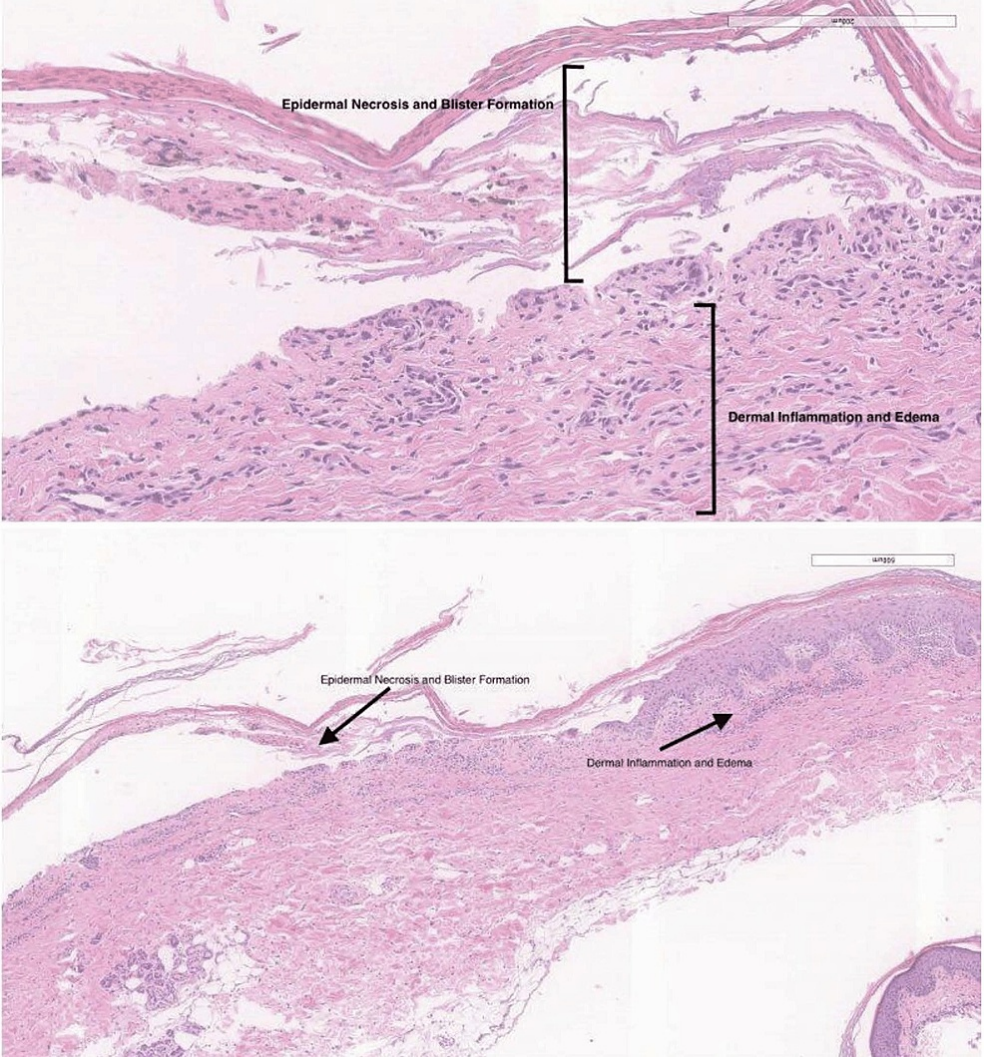
Two frozen-section shave biopsies from a dusky erythematous macular area of impending blister formation on the left proximal forearm showing intraepidermal clefting with areas of full thickness epidermal necrosis, characteristic of Stevens-Johnson syndrome or toxic epidermal necrolysis.

**Figure 4:**
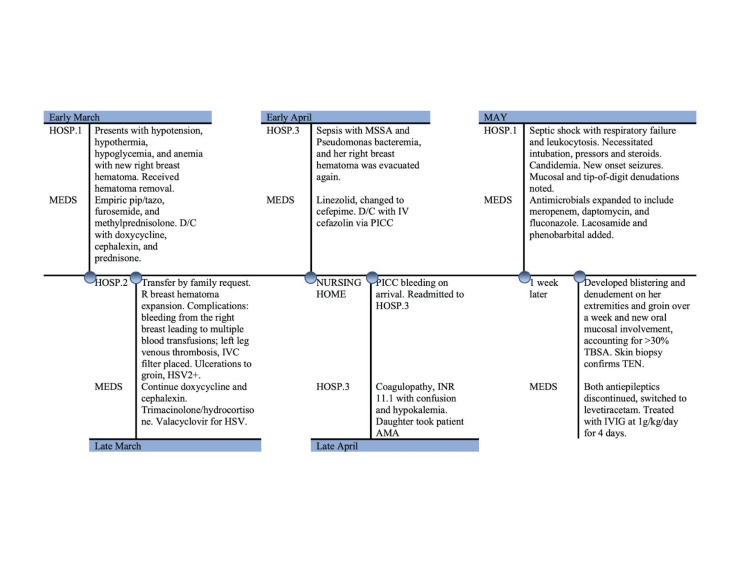
Chronological course of TEN presentation, diagnosis, treatment, and outcomes. D/C, discharged; HSV, human simplex virus; IVC, inferior vena cava; IVIG, intravenous immunoglobulin; MSSA, methicillin-sensitive staphylococcus aureus; PICC, peripherally inserted central catheter; R, right-sided; TBSA, total body surface area; TEN, toxic epidermal necrolysis. Figure credits: Rebekah Lantz.

Nikolsky and Asboe-Hansen signs were positive, suggestive of a differential for Staphylococcal scalded skin syndrome (SSS), toxic shock syndrome (TSS), immunobullous eruption, or SJS. Skin shave biopsies from the left proximal and distal forearm were evaluated by the pathologist and exhibited changes consistent with SJS (Figure 3). Score of TEN (SCORTEN) was 2 for age and amount of TBSA, with a predicted 12.1% mortality rate. The clinicopathologic impression was TEN with lesions covering >30% TBSA. Subsequently, both antiepileptics were discontinued and changed to levetiracetam, which was associated with fewer side effects. From a chronological standpoint, cephalosporins were presumed as the likely etiology by our dermatology specialists although multiple agents may have been the cause or contributed as well as her HSV infection and underlying dermatologic condition of atopic dermatitis. She was treated successfully with a course of intravenous immunoglobulin (IVIG) at 1 g/kg per day for four days. Figure 4 delineates the chronology of a more detailed clinical course.

Unfortunately, the patient suffered status epilepticus at the long-term acute care hospital (LTACH) to which she had been discharged, with the development of further viral (influenza A) and bacterial infections (acinetobacter and methicillin-resistant Staphylococcus aureus [MRSA] bacteremia) so that treatment was expanded with further antimicrobials, further antiepileptic drugs (AEDs), and a blood transfusion for anemia. She remained ventilator dependent, hemodialysis dependent, and on tube feeds, with an overall progressive decline so that there were further discussions with family and eventual change to do-not-resuscitate (DNR) status. Further records are not available outside the facility, but it is known that she died nine months after the onset of all symptoms at the LTACH facility.

## Discussion

This case report presents several important clinical pearls. It is crucial to recognize and understand warning skin findings, potential contributing medications, infections, underlying disease predispositions, and other risk factors for SJS/TEN. One of the strongest risk factors for SJS/TEN is an increasing number of chronic comorbid conditions [4]. In this patient’s complex clinical course, the odds of severe morbidity and mortality were increased significantly due to her history of autoimmune and inflammatory conditions as well as her critical conditions that required multiple hospitalizations. Additionally, multiple stays within different hospital systems can contribute to delayed diagnosis and unclear predisposing factors, including medications, comorbid illnesses, and infections. The initial symptoms of SJS/TEN are reportedly nonspecific and can precede cutaneous manifestations by a few days, typically one to three days in one-third of cases [5]. These symptoms may include painful mucous membranes, stinging eyes, headache, rhinitis, cough, sore throat, and myalgias [6]. Notably, these symptoms are difficult to discern in critically ill and encephalopathic patients. This makes early recognition and treatment challenging, which increases the risks of erroneous initial diagnosis and delayed management of a medical emergency.

Due to our patient’s multiple acute and chronic medical issues, she was exposed to numerous new and chronic medications at various doses, including high-risk medications such as antibiotics and antiseizure agents. In our patient, acute kidney injury may be responsible for her rapid decline and is an independent risk factor for increased mortality in TEN [7]. It is well documented in the literature that antimicrobials such as cephalosporins and fluoroquinolones can induce SJS and TEN [8-11], which our patient was receiving. Although high-risk agents were stopped immediately upon her diagnosis, SJS/TEN can occur a few days after the offending drug has been withdrawn in the case of long-half-life drugs. Moreover, in some cases where the same drug is reintroduced, also known as *rechallenge*, the disease appears more rapidly within hours [8]. The interplay of critical illnesses in a multidrug regimen led to a complex discovery of our patient’s dermatologic manifestation. The onset of SJS/TEN in our patient may likely have been confounded by her comorbidities, newfound infections, and extensive pharmacologic treatment.

It is important to note other predisposing factors in patients of African American descent and other skin-of-color races. Current understanding of the pathogenesis of drug hypersensitivity that leads to SJS/TEN includes T-cell-mediated cytotoxicity, genetic linkage with HLA genes, and other cytotoxicity mechanisms [12]. Lu et al. studied the racial disparities in the risk of SJS due to specific HLA alleles that affect urate-lowering drugs such as allopurinol [13]. This study indicated that Asians (27%) and Blacks (26%) are at a substantially higher risk of SJS/TEN due to their high frequency of carrying the HLA-B*5801 allele [13]. Another study revealed that non-whites such as Asians and African Americans had a significantly higher risk for SJS/TEN compared to Caucasians [4].

While polymorphism genetics such as HLA may contribute to drug reactions, less is known about the nongenetic causes of SJS/TEN. In their study, Okamoto-Uchida et al. [14] mentioned that concurrent infection and pathology significantly escalate the timing and worse condition of progression to SJS/TEN in both dermatological and ophthalmological phenotypes. In the case study of this patient, a single underlying etiology is unclear but a progressive infectious course contributed to her overall critical presentations.

Medical textbooks and education materials have an inherent lack of skin-of-color representation, which can contribute to delayed recognition by the healthcare team [15]. Dutt et al. in 2020 showcased African American patients who were evaluated two to three times for mucocutaneous lesions before being admitted for a diagnosis of SJS [16]. We can improve outcomes for patients by recognizing disparities toward patients of darker skin and that there are varying colors and morphologies with dermatologic conditions. This case contributes to literature advancement by discussing TEN findings in the skin of color, skin-of-color risk factors such as underlying genetic predispositions, and initial skin changes that may be telling signs of SJS/TEN in critically ill patients.

Chat Generative Pre-trained Transformer (ChatGPT)

This case report was written with the assistance of ChatGPT (Version January 30, 2023; OpenAI LP, OpenAI Inc., San Francisco, CA, USA). ChatGPT is a form of artificial intelligence (AI) language model that uses deep learning to generate seemingly informative text responses. It incorporates books, articles, and websites and learns patterns and relationships from the dataset to output human-like interactions with a person.

**Figure 5:**
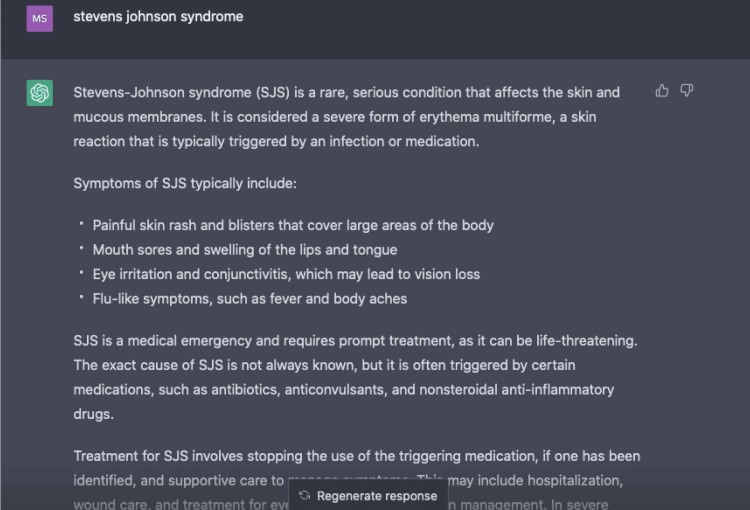
ChatGPT screen and response. ChatGPT, Chat Generative Pre-trained Transformer

**Figure 6:**
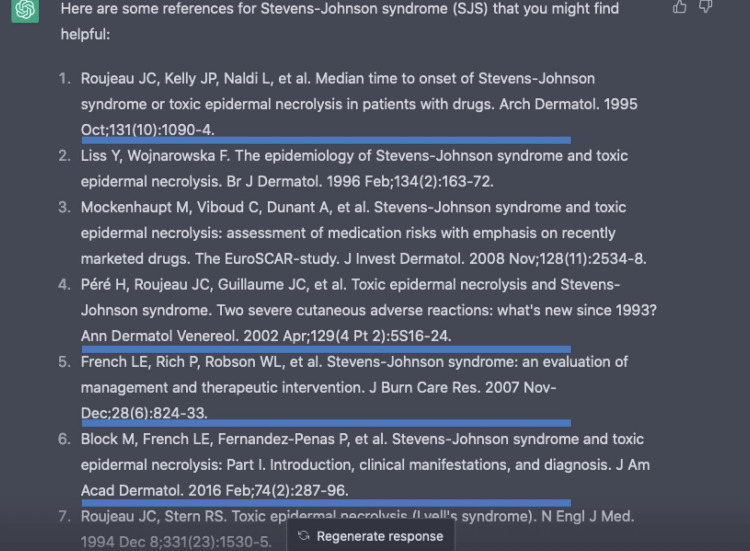
ChatGPT interface in which seven references were provided of which 1, 4, 5, and 6 were nonexistent. ChatGPT, Chat Generative Pre-trained Transformer

Currently free through OpenAI's website (https://openai.com/), we appreciated the use of ChatGPT to gain background information about disease descriptions, drug interactions, and references to launch our study. We fact-checked with existing literature to ensure accuracy, and the wording and model response is shown in Figure 5. There were important downtimes when ChatGPT was at full capacity and unable to sign in or process work. We requested references for our topic, which were perfectly formatted. However, upon fact-checking, of the seven references provided, four were nonexistent, as underlined in Figure 6. Additionally, most of the references were 10 years or longer from publication, whereas the medical field is constantly garnering new evidence and changing. More recent literature is preferred in citations, and clear errors in citations cannot be overlooked.

While there are some benefits to using AI such as ChatGPT, including information gathering and suggested references, it has several profound limits: there is still the need to fact-check for accuracy, and as the majority of references provided did not exist in separate search history, so it is unclear from where these were generated. It is our impression that ChatGPT use is beneficial in some regards, but its limitations should also be well understood and cannot replace human research skills and independent investigation.

## Conclusions

SJS and TEM are life-threatening dermatologic emergencies. Early recognition can significantly improve the prognosis. This case conveys the importance of close monitoring of the critically ill patient, including identifying skin and mucosal surfaces for changes indicative of an early drug reaction. High-risk medications and suspected causative agents should be discontinued immediately as well as underlying infections treated expeditiously with the guidance of a multimodal team of prescribing physicians and specialists. In patients of darker skin, providers should have an understanding of underlying predispositions and differences in cutaneous drug reaction morphology compared to lighter skin tones. Our goal is to contribute to current literature on SJS/TEN in patients with multiple risk factors, including complicated medical conditions, genetic predispositions, and concomitant conditions that may lead to a more critical presentation and course. We also hope to broaden the literature on recognizing life-threatening dermatologic conditions in patients with darker skin. Also when using AI such as ChatGPT, care should be taken to fact-check information and reference materials.
